# Mental health and loneliness in the German general population during the COVID-19 pandemic compared to a representative pre-pandemic assessment

**DOI:** 10.1038/s41598-021-94434-8

**Published:** 2021-07-22

**Authors:** Manfred E. Beutel, Nora Hettich, Mareike Ernst, Gabriele Schmutzer, Ana N. Tibubos, Elmar Braehler

**Affiliations:** 1 Department for Psychosomatic Medicine and Psychotherapy, University Medical Center of the Johannes Gutenberg University Mainz, Untere Zahlbacher Str. 8, 55131 Mainz, Germany; 2 Behavioral Medicine Research Unit, Integrated Research and Treatment Center Adiposity Diseases, Clinic and Polyclinic for Psychosomatic Medicine and Psychotherapy, Leipzig University Medical Center, Semmelweisstr. 10, 04103 Leipzig, Germany

**Keywords:** Psychology, Risk factors

## Abstract

During the pandemic, the extent of subsequent mental health strains is an important issue. A representative face-to-face survey was conducted to assess mental health consequences in the general population and to identify mental health risk factors. In a representative German sample (*N* = 2,503), we assessed depression and anxiety symptoms by the PHQ-4 and loneliness by a validated item. An earlier survey (2018) which used the same methods and had comparable response rates served as comparison. Scores of depression and anxiety symptoms increased from an average of 0.89 (*SD* = 1.21) and 0.77 (*SD* = 1.17) in 2018 to 1.14 (*SD* = 1.23) and 1.05 (*SD* = 1.31) in 2020. Loneliness did not increase (*M* = 1.35, *SD* = 0.68 in 2018; *M* = 1.38, *SD* = 0.78 in 2020), affecting about one in four participants to some degree. Younger participants and women were most likely to report depression, anxiety, and loneliness. As in the previous survey, social inequality factors contributed to distress and loneliness. The small overall increase of distress was consistent with recent German panel studies. In future studies and mental health interventions female sex, younger age, and socioeconomic disparities need to be considered as vulnerability factors for distress.

## Introduction

The international public health crisis of the COVID-19 pandemic poses health threats of unprecedented magnitude and may increase the risk for mental disorders^1–3^. In a frequently quoted online survey of 1,210 participants from 194 Chinese cities, 54% of the participants reported moderate to severe distress, 29% reported anxiety and 17% depression symptoms^4^. In a German online survey conducted during the first lockdown, between March 27th and April 06th, 2020, 25% of the participants scored above the cut-offs for anxiety (GAD-2) and for depression (PHQ-2). They reported spending an average of almost five hours per day thinking about the pandemic, which exacerbated their stress^5^. A German cross-sectional online survey which ran from March to May 2020 with over 15,000 participants reported increased generalized anxiety (GAD-7) in 44.9%, depression in 14.3% (PHQ-2) and COVID-19-related fear in 59% of the participants^6^. Based on a total of 43 trials mostly from Asia and Europe, according to a recent meta-analysis, anxiety had tripled at the current rate of 25%, compared to previous rates of 7.3%^7^. Another meta-analysis even found that 31.9% of 63,439 people reported anxiety and 33.7% of 44,531 people depression symptoms^8^. However, the vast majority of studies relied on self-selection of participants by online advertisement and snowball effects and thereby excluded citizens without regular access to the internet^9^. Exemplarily, in one online survey, participants were predominantly female, of young age, highly educated, and health care staff was overrepresented^5^.

A large British online panel with over 60,000 participants which had started in March 2020 reported higher levels of loneliness based on the three-item UCLA Loneliness Scale with 32.5% feeling sometimes and 18.3% often lonely, compared to figures from previous panels of 28.6%, respectively 8.5%^10^. Recently, a longitudinal household panel of the German general population of 4,577 participants during the lockdown period (from March 31st to July 28th, 2020) found a small increase of anxiety and depression (PHQ-4) compared to the previous year. However, scores were comparable to the 2016 survey. Compared to a previous survey in 2017, loneliness was increased by about one standard deviation and was most prevalent among younger and female participants^11^. Based on 113,928 participants, a recent report from the German National Cohort found a moderate increase of clinically relevant depression (PHQ-9: from 6.4 to 8.8%) and anxiety symptoms (GAD-7) from 4.3 to 5.7%^12^.

As the pandemic affects the entire population, it is crucial to identify risk factors for mental health problems and need for support^13^. In the studies summarized above, female sex, younger age, student status and living in Asia vs. other continents were risk factors for heightened anxiety, depression, and loneliness^4,6,8,10–12,14^. Distress was higher in regions of Germany with higher infection rates^12^. Additional risk factors for loneliness were low household income and living alone^10^. Health risks related to COVID-19 morbidity and mortality have been shaped by socio-economic inequality, disproportionally affecting marginalized groups^15^. Yet, indicators for health and mental health disparities such as low income, unemployment or migration background have hardly been taken into account in the reported studies.

This paper contributes to the important issue to what degree the pandemic has interfered with the mental health of the general population and identifies underlying risk factors. Using self-report scales, we assessed depression and anxiety symptoms with the established PHQ-4^16^ and loneliness with a validated item^17^ in a representative face-to-face survey from May 2nd to June 29th, 2020. At this time, lockdown measures were successively being reduced, however, most schools and childcare facilities remained closed. We used a representative cohort from 2018 which had been drawn by the same methods as pre-pandemic comparison. We aimed to answer the following research questions:

1. What is the level of depression and anxiety symptoms, and loneliness in the general population after the first wave of the COVID-19 pandemic compared to previous mental health data?
2. How are sex, age, and socio-demographic factors such as low income, unemployment, migration status, and partnership related to distress and loneliness?

## Results

### Depression, anxiety, and loneliness

There was a significant overall increase of depression and anxiety symptoms in 2020 as compared to 2018 with a small effect size of 0.19^16^. While the sum scores of symptoms were slightly lower than the population norms in the 2018 assessment, they exceeded the norms in 2020^16^. In 2020, 11.6% of participants scored above the cut-off for depression, compared to 8.3% in 2018. A total of 11.1% scored above the cut-off for anxiety, compared to 7.5% in 2018. For loneliness, no significant change between 2018 and 2020 was found. In 2020, some degree of loneliness was reported by 23.1% of the participants as compared to 24.5% in 2018. The data is presented in Table 1.

**Table 1:** Comparisons of depression and anxiety symptoms, and loneliness between 2018 and 2020 for all participants, women, and men.

	Survey 2018 (*N* = 2,516)		Survey 2020 (*N* = 2,503)		*d*^*1*^
	*M*	*SD*	*M*	*SD*	
**PHQ-2**	.89	1.21	1.14	1.23	0.19
Men	.86	1.22	1.03	1.17	0.14
Women	.92	1.20	1.24	1.27	0.26
**GAD-2**	.77	1.17	1.05	1.31	0.19
Men	.68	1.12	.89	1.22	0.18
Women	.85	1.20	1.19	1.37	0.26
**Loneliness**	1.35	.68	1.38	.78	0.04
Men	1.32	.64	1.31	.71	0.02
Women	1.38	.72	1.45	.84	0.09

	N	%	N	%	*p*
**PHQ-2 > = 3**	209	8.4	289	11.6	***
Men	101	8.9	114	9.8	n.s
Women	108	7.9	175	13.3	***
**GAD-2 > = 3**	187	7.5	276	11.1	***
Men	72	6.3	101	8.7	*
Women	115	8.4	175	13.3	***
**Loneliness**^2^	608	24.5	573	23.1	n.s
Men	261	23.2	224	19.3	*
Women	347	25.7	348	26.3	n.s

Norm scores: PHQ-2: 0.94 (1.20), GAD-2: 0.82 (1.10)^16^; ^1^ Cohen’s d; ^2^ at least some degree; Chi^2^: *p < 0.05, **p < 0.01, ***p < 0.001.

**Figure 1:**
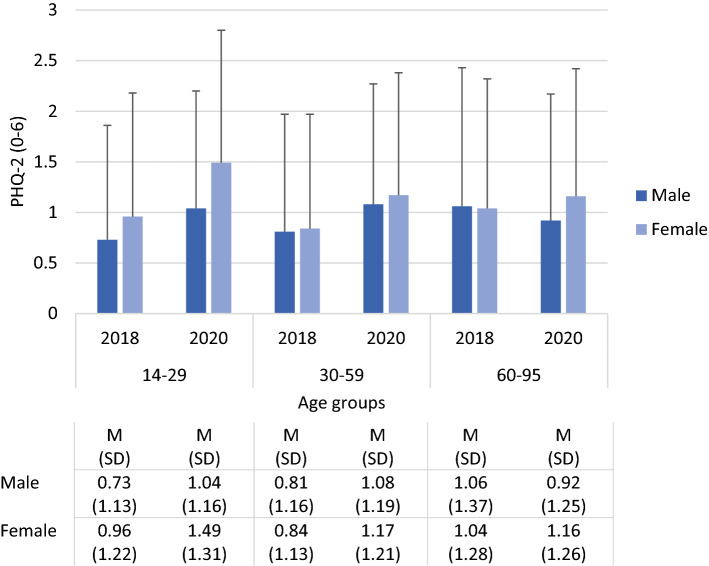
Depression scores in 2018 and 2020 by age group and sex. Analysis of variance: Survey (2018, 2020) by sex (male, female) by age group (14–29, 30–59, 60–95); F survey (df = 1) = 52.18, p < .001; F Sex (df = 1) = 14.36, *p* < .001; F age group (df = 2) = 3.31, *p* = .037; F survey by sex (df = 1) = 4.61, *p* = .03; F survey by age group (df = 2) = 15.11, *p* < .001; F sex by age group (df = 2) = 5.13, *p* = .006.

Figure 1 presents the mean level of depression symptoms in 2018 and 2020 stratified by age group and sex. As statistically tested using ANOVA, scores were higher in women vs. in men (sex), in 2020 vs. in 2018 (survey), and in younger vs. in older participants (age group). We also observed statistically significant interactions, i.e., scores increased more in women than in men (survey by sex), in the younger vs. older age groups (survey by age group), and in younger vs. older women (sex by age group). The largest increase was found in young women aged 14 to 29 years.

**Figure 2:**
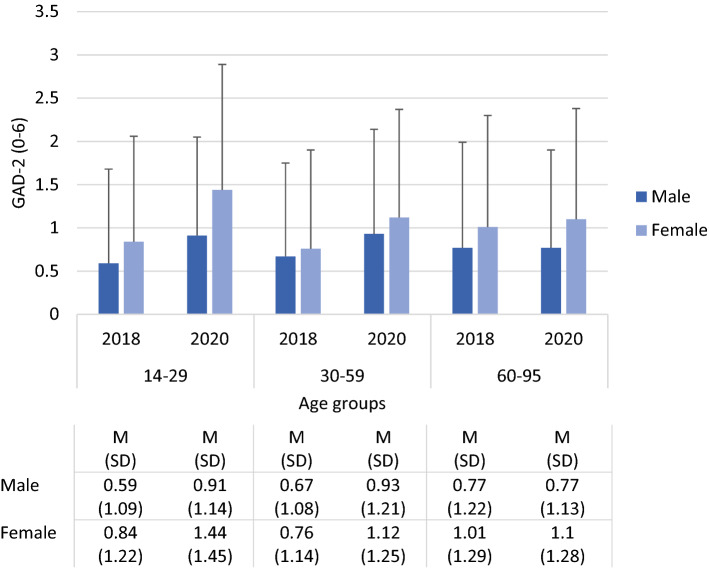
Anxiety scores in 2018 and 2020 by age group and sex. Analysis of variance: Survey (2018, 2020) by sex (male, female) by age group (14–29, 30–59, 60–95); F survey (df = 1) = 62.21, *p* < .001; F Sex (df = 1) = 44.02, *p* < .001; F age group (df = 2) = 2.27, *p* = .10; F survey by sex (df = 1) = 4.06, *p* = .04; F survey by age group (df = 2) = 13.41, *p* < .001; F sex by age group (df = 2) = 6.59, *p* = .013.

Figure 2 presents the anxiety symptom scores in 2018 and 2020 stratified by age group and sex. As determined by the ANOVA, scores were higher in women vs. in men (sex) and in 2020 vs. in 2018 (survey). Again, there were significant interactions, i.e., sum scores increased more in women than in men (survey by sex), in the younger vs. the older age groups (survey by age group) and in younger vs. in older women (sex by age group). The largest increase was found in young women aged 14 to 29 years.

**Figure 3:**
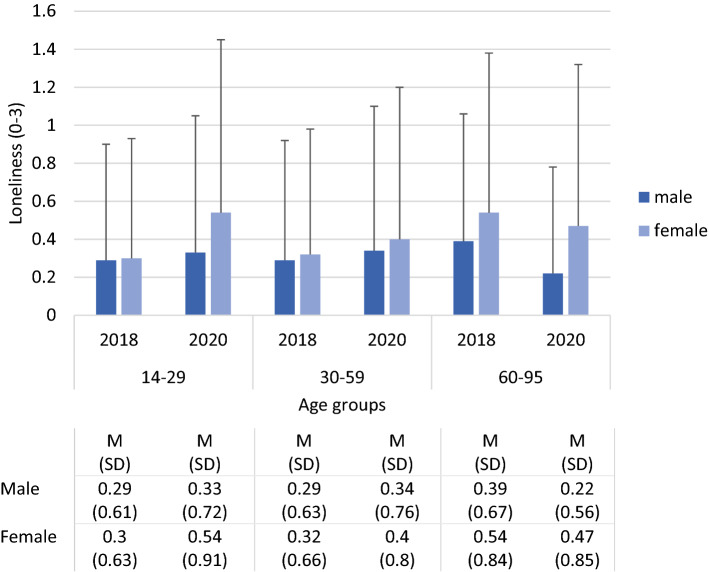
Loneliness scores in 2018 and 2020 by age group and sex. Analysis of variance: Survey (2018, 2020) by sex (male, female) by age group (14–29, 30–59, 60–95); F survey (df = 1) = 3.16, *p* = .076; F Sex (df = 1) = 22.75, *p* < .001; F age group (df = 2) = 4.99, *p* = .007; F survey by sex (df = 1) = 4.16, *p* = .041; F survey by age group (df = 2) = 10.64, *p* < .001; F sex by age group (df = 2) = 5.14, *p* = .006.

Figure 3 presents the loneliness scores in 2018 and 2020 according to age group and sex. As determined by the ANOVA, scores were higher in women vs. in men (sex) and in older vs. in younger participants (age group). There was only a trend for an increase in 2020 vs. 2018 (survey). As the interaction effects indicated, scores increased more in women than in men (survey by sex), in the younger vs. in the older age groups (survey by age group) and in younger vs. in older women (sex by age group). The largest increase was found in young women aged 14 to 29 years.

### Risk factors for depression, anxiety, and loneliness

The multiple linear regression analyses are presented in Table 2. Regarding depression symptoms, there was a shift in the most relevant statistical predictors from 2018 to 2020. In 2018, unemployment was the strongest predictor, followed by low household income, higher age, migration background, and lack of a partnership. In 2020, low household income and unemployment were the strongest predictors, followed by female sex, lack of a partner, and migration background. However, age no longer had a statistically significant association with depression symptoms, while female sex was related to higher levels of depression symptoms. Education was not statistically predictive in both surveys.

**Table 2:** Determinants of depression and anxiety symptoms, and loneliness in 2018 and 2020.

	Depression		Anxiety		Loneliness	
	2018	2020	2018	2020	2018	2020
	Beta	Beta	Beta	Beta	Beta	Beta
Lack of Partnership	.041*	.062 **	.055**	.029	.225***	.151 ***
Age	.101 ***	.000	.097 ***	− .011	.140***	.008
Unemployment	.174 ***	.112 ***	.151 ***	.134***	.100 ***	.069 **
Household income	− .104***	− .133 ***	− .099***	− .116 ***	− .095***	− .096 ***
Migration background	.090 ***	.059 **	.081 ***	.066 **	.056 **	.016
Female sex	.018	.076 ***	.063 **	.106 ***	.030	.085 ***
High education	− .007	− .007	.029	− .013	.023	− .018
Adj. R^2^	.07 ***	.06***	.06***	.06***	.11***	.06***

*p < 0.05, **p < 0.01, ***p < 0.001.

Regarding anxiety symptoms, a similar pattern was found. In 2018, unemployment was the strongest statistical predictor, followed by low household income, higher age, migration background, female sex, and lack of a partnership. In 2020, low household income and unemployment were the strongest predictors, followed by female sex and migration background. Age and partnership were no longer statistically predictive. Education had no relevant associations with anxiety symptoms in both surveys.

Regarding loneliness, in 2018 the lack of a partnership was the strongest predictor, followed by higher age, unemployment, low household income, and migration background. In 2020, low household income and the lack of a partnership were the strongest predictors, followed by female sex and unemployment. Unlike 2018, age and migration background were no longer statistically predictive, but female sex was associated with higher levels of loneliness. The level of education was not related to loneliness within this model in both surveys.

## Discussion

The present study investigated changes in mental distress and loneliness in conjunction with the COVID-19 pandemic using two representative German survey samples. In the second survey, conducted in spring 2020 after initial lockdown measures had been eased, depression and anxiety symptoms, but not loneliness was significantly increased compared to the pre-pandemic assessment from 2018. However, the differences were small. In 2020, 11.6% of participants fulfilled the criteria for clinically significant depression symptoms (vs. 8.3% in 2018) and 11.1% scored above the cut-off for anxiety symptoms (vs. 7.5% in 2018).

These findings contradict previous, internet-based, international, and German surveys, which found exceedingly high levels of depression and anxiety affecting 25% to 35% of participants during the pandemic^4–6,8,14^. Our findings regarding depression and anxiety were more consistent with two recent German longitudinal panel studies reporting small increases of distress^11,12^. However, based on a single and validated item to assess loneliness, we only found an increase in young participants while other studies reported a significant increase of loneliness for all participants^11^. As findings from German online surveys were comparable to other international surveys, we would presume that cross-sectional online convenience samples are likely biased evoking responses from young and educated subgroups, and from those most preoccupied with the pandemic.

In order to adapt mental health care to the needs of the population, it is crucial to identify those most distressed by the pandemic^13^. Our study showed that risk factors pertain to different domains of life and that they interact: Distress and loneliness increased most in young participants and in women. In the full regression model, consistent risk factors were unemployment, low household income, and female sex. Lack of a partnership was an additional risk factor for depression and loneliness, and migration background was associated with depression and anxiety. In the 2018 survey, higher age had been predictive of distress and loneliness. However, due to the increases in younger groups, age was no longer a predictor in 2020.

When worldwide lockdown measures were implemented, there were widespread concerns that older persons, who were most vulnerable with regard to morbidity and mortality in case of an infection with the virus, would additionally suffer from loneliness and distress^18,19^. Contrary to the expectations, in 2020 distress and loneliness had not increased in older individuals. However, particularly vulnerable subgroups of older people who are confined to nursing homes or disabled by chronic disease or frailty might be less likely to participate in any nationwide survey and be therefore underrepresented. The overall shifts we found were due to an increase of distress and loneliness in younger age groups, particularly those under 30 years. Carrying the lowest risk of severe disease, this age group has initially drawn little attention regarding mental health outcomes in the COVID-19 literature^20^. In this age group, however, many of the risk factors we assessed, apply, e.g., the lack of a partnership, and low household income. As previous research has argued^21^, young adulthood is a period with a high incidence of mental disorders and with strong needs for social relationships, activities with peers and large-scale events such as parties, sports events, movies, and concerts. These occasions for socializing have been completely prohibited, even beyond the strictest lockdown measures.

It is an intriguing question why adverse effects were seen in women more than in men, particularly in young women. One explanation is that many of them likely had high risks for infection due to their field of work (e.g., as health care professionals, salespersons, and waitresses). Moreover, according to recent German and US surveys, women have been overly burdened by conflicts between child care and work as schools and nurseries were closed^21^. Mothers were four to five times more likely to have reduced their working hours compared to fathers^22^. The combination of heightened childcare demands, reduction of working hours, and employment losses in heavily affected sectors, such as restaurants, has led economists to predict a widening of the gender wage gap^23^.

While the proportion of explained variance was small, we observed that low income, unemployment and migration status diminished mental health. The pandemic thus aggravates social inequity as especially groups who already were at risk for mental health problems (e.g., individuals with migration background, without employment, and with a low income) suffer the most from both the measures aimed at its mitigation and its socio-economic consequences. They are more likely to be infected and, if infected, to be hospitalized. , while Economic losses and social isolation might further increase distress and loneliness^24,25^. Our findings are consistent with the growing concerns that existing inequalities in access to mental health care may become more pronounced as a consequence of the pandemic^13^.

Our data have implications for a shift in the prevention of mental disorders to particularly burdened individuals. In the ongoing pandemic, our data indicate the necessity to support subgroups such as young women, for example, at a society-level through political decisions that promote the compatibility of work and family life and at a person-level through web- or app-based interventions. There is also a need to improve opportunities to reach individuals of low socio-economic status and people with migration backgrounds.

Strengths of the study were the availability of representative surveys conducted using identical methods and measures at two time points. Unlike previous representative surveys^11^, we did not switch from in-person to telephone interviews, which might have biased the findings. We replicated earlier surveys while adhering to the health and sanitation requirements of the pandemic. As the rate of participation shows, overall acceptance by participants was comparable to previous surveys. Yet, as in the other surveys, our interpretations are limited by the fact that we collected independent samples. Therefore, it is only possible to report association between groups and time points without being able to draw conclusions about causality. Our findings need to be replicated with longitudinal panel data.

In our study, we did not assess directly pandemic-related threats to mental health as, for example, fear of COVID-19, specific stressors (e.g., loss of loved ones to COVID-19), or barriers to social participation (e.g., internet access). Hence, neither the relevance of those aspects for the general population’s well-being nor distinct risk factors for these domains can be derived from our findings. They need to be investigated in future large-scale, population-based studies.

Studying the mental health sequelae of the ongoing COVID-19-pandemic is a process that needs to take the context of the current state of the pandemic into account. Our survey period extended beyond the initial lockdown in Germany, which means that our results do not reflect only the situation during the full lockdown. Mental distress, loneliness, or stress may have already been alleviated by the time of assessment as participants scored higher on some measures compared to the pre-pandemic assessment. However, while the number of active COVID-19 cases had decreased, restrictive measures such as the closure of schools, universities, and childcare, as well as social distancing recommendations and bans on social gatherings, still applied. In future studies, it will be important to adjust and report the timing of surveys to reflect the dynamics of the pandemic and the measures taken as well as possible.

## Methods

### Study design and participants

In cooperation with the demography research institute USUMA Berlin, data of two representative samples of the German population (age 14–95) were collected in 2018 and 2020, employing a random route approach. First, 258 German regional areas were predefined using the reference system for representative studies in Germany provided by the ADM-Sampling-System which offers a sampling frame covering all populated areas of Germany and allowing for the drawing of representative face-to-face samples^26^. Afterwards, the target households within these regional areas were selected following a random route procedure. For multi-person households, one person was randomly selected by means of the Kish grid technique method for selecting members of a household to be interviewed^27^.

Assessments in 2020 were conducted from May 2nd to June 29th, 2020 when lockdown measures to combat the COVID-19 pandemic were successively reduced. Face-to-face interviews were carried out following the hygiene regulations (wearing facial masks and keeping physical distance). Questionnaires were handed out to be filled out by the participants. A total of 2,503 participants took part, representing 46.5% of 5,418 households addressed. The comparison group consists of interview data collected from May to July 2018 which comprises 2,516 participants, representing 62% of 4,091 households addressed. By comparisons with the Federal Statistical Office, both samples proved to be representative for the German general population regarding age, sex, and education.

The study was conducted in accordance with the Declaration of Helsinki and fulfilled the ethical guidelines of the International Code of Marketing and Social Research Practice of the International Chamber of Commerce and the European Society of Opinion and Marketing Research. Prior to being carried out, the surveys’ procedures and contents were approved by the Ethics Committee of the Medical Faculty of the University of Leipzig. Anonymity in responses was guaranteed and the informed consent was obtained from all respondents, who indicated willingness to take part in the study. In line with the guidelines of the Working Group German Marketing Institutes and Social Research Practice, it is generally assumed that respondents aged 14 or above are capable of consenting to the use of the information provided in survey research. Due to the nature of face-to-face studies, one parent is usually informed about the content of the study and the selection procedure before the survey begins. However, this is not obligatory for the participation of the adolescent. To be eligible for survey inclusion, participants had to be at least 14 years of age and have sufficient German language skills. Prospective subjects were told that the study was about psychological health and well-being.

Table 3 gives an overview of the study participants. The mean age of the participants in the 2020 survey was 46 years. Due to slightly higher proportions of the age group 14 to 29 and lower proportions of the age group 60 to 95, participants were slightly younger than in the 2018 survey (*M* = 48 years). Additionally, the mean income and the number of participants holding at least a high school diploma were higher in the 2020 survey. In 2020, the characteristics of sex (47% were male), migrant background (16% had a migrant background), partnership (60% lived in a partnership), and unemployment (5.9% were unemployed) did not differ significantly from the 2018 survey data.

**Table 3:** Study participants of the surveys 2018 and 2020.

		Survey 2018 (*N* = 2,516)		Survey 2020 (*N* = 2,503)		*p*
		*N*	%	*N*	%	
Age group	14–29	466	18.5	563	22.5	
	30–59	1,345	53.5	1,317	52.6	
	60–95	705	28.0	623	24.9	
Sex	Male	1,144	45.5	1,173	46.9	n.s
	Female	1,372	54.5	1,329^1^	53.1	
Partnership	Yes	1,468	59.3	1,443	59.9	n.s
Unemployment	Yes	135	5.4	146	5.9	n.s
Migration background	Yes	378	15.0	394	15.7	n.s
High education^2^	Yes	513	20.4	763	30.6	***

		Mean	*SD*	Mean	*SD*	
Age	Years	48.03	17.57	45.99	17.77	***
Income	Euro	1,827.01	929.68	2,026.10	1,087.88	***

^1^: *N* = 1 diverse; ^2^: at least high school diploma; *p < 0.05, **p < 0.01, ***p < 0.001.

### Measures

Socio-demographic information such as age, sex, unemployment, migration status, and equivalence household income were assessed in face-to-face interviews with all respondents.

Self-report questionnaires assessed depression and general anxiety (PHQ-4). The two-item questionnaire PHQ-2 measures anhedonia (“Little interest or pleasure in doing things”) and depressed mood (“Feeling down, depressed or hopeless”) over the past two weeks^28^. Its sum score ranges from 0 to 6. Scores of 3 and above indicate a depressive disorder with a sensitivity of 79% and a specificity of 86%. The PHQ-2 shows a high reliability of α = 0.83^16,29^. In the present sample the internal consistency was good in 2018 (α = 0.82) and acceptable in 2020 (α = 0.71).

Symptoms of anxiety were measured with the two-item questionnaire GAD-2 which assesses being bothered by “feeling nervous, anxious, or on edge” and “not being able to stop or control worrying” over the last 2 weeks^30^. The GAD-2 score ranges from 0 to 6. Scores of 3 and above indicate an anxiety disorder (e.g., generalized anxiety disorder, social phobia, or panic disorder) with a sensitivity of 65% and a specificity of 88%. The reliability of the GAD-2 was acceptable with α = 0.75^16,31^. In the present sample, the internal consistency was good in 2018 (α = 0.81) and acceptable in 2020 (α = 0.76).

Loneliness was assessed using the single item: “Does the following apply to you, and if so, how much of a burden does it place on you? Frequent loneliness, too few contacts."^17^. This item was rated as “no, does not apply”, “yes, it applies, but I do not suffer from it”, “yes, it applies, and I suffer slightly”, “yes, it applies, and I suffer moderately”, or “yes, it applies, and I suffer strongly”. For the analyses “no, does not apply” and “yes, it applies, but I do not suffer from it” were combined to the score 0 = no loneliness. “Yes, it applies, and I suffer slightly” was coded as 1, “yes, it applies, and I suffer moderately” as 2, and “yes, it applies, and I suffer strongly” as 3. Thus, the score ranges from 0 to 3 with a higher score indicating a higher level of loneliness.

### Statistical analysis

One-way multifactorial ANOVAs with post hoc analyses (Scheffé method) were conducted for group comparisons using demographic variables (age group and sex) as well as survey as independent, and depression, anxiety, loneliness as dependent variables. Chi^2^ was used as a nonparametric test. Effect sizes correspond to Cohen’s *d*. Separate multivariable regression analyses of depression, anxiety, and loneliness were carried out for the two surveys to determine the statistical relevance of the predictors age, sex, migration background, partnership, education, and unemployment. Significance for statistical tests was set at *p* < 0.05 (two-sided). All analyses were conducted using SPSS, Version 25.0^32^.

## Data Availability

For approved reasons, some access restrictions apply to the data underlying these findings. Data sets contain identifying participant information, which is not suitable for public deposition. Access to the local database is available upon request to the corresponding author.
